# Supplementary material for: MetaRibo-Seq measures translation in microbiomes
Source: Nat Commun. 2020 Jun 29;11:3268. doi: 10.1038/s41467-020-17081-z (PMC7324362; doi:10.1038/s41467-020-17081-z)
Supplement: Supplementary file 10 — Supplementary Data 7 [file 41467_2020_17081_MOESM10_ESM.zip › File2/Confidence_High_Taxonomy/70135_out.krona.html]

Javascript must be enabled to view this page.

members
magnitude
magnitudeUnassigned
count
unassigned
taxon
rank

70135\_out

4

superkingdom
4
2

phylum
1239
2

class
2
91061

2
186826
order

1300
2
family

2
1301
genus


SRS074676\_contig\_number\_contig-100\_367.62725
species
1
1318


SRS056537\_contig\_number\_44766
species
1305
1

1224
2
phylum

class
2
1236

135625
2
order

2
712
family

724
2
genus

729
2
species

SRS015797\_contig\_number\_contig-100\_18989.18989SRS064014\_contig\_number\_9802
